# Development and validation of an HILIC–MS/MS method by one-step precipitation for chloroquine in miniature pig plasma

**DOI:** 10.4155/bio-2015-0032

**Published:** 2016-05-23

**Authors:** Zhan-Zhang Wang, Hao-Yang Lu, De-Wei Shang, Xiao-Jia Ni, Ming Zhang, Yu-Guan Wen

**Affiliations:** 1 The Affiliated Brain Hospital of Guangzhou Medical University (Guangzhou Huiai Hospital), Guangzhou 510370, China

**Keywords:** chloroquine, HILIC, LC–MS/MS

## Abstract

**Background:**

Quantification of polar compounds such as chloroquine by revered-phase LC is a challenge because of poor retention and silanol interactions with stationary phase. Strong ion-pairing reagents added to mobile phases to improve reversed-phase retention and improve peak shape can be harmful for MS.

**Results:**

This new approach provides a rapid and sensitive method for the detection of chloroquine using hydrophilic interaction LC coupled to MS/MS (HILIC–MS/MS). Ammonium formate and formic acid were added to mobile phase to attain good peak shapes and the salified chloroquine as well retained in an HILIC column. Linearity, intra- and inter-day precision, accuracy, recovery, matrix effect and stability were evaluated during the validation process.

**Conclusion:**

The validated method has been successfully used in a PK study in miniature pigs, and paves way for future development.

**Figure 1.**
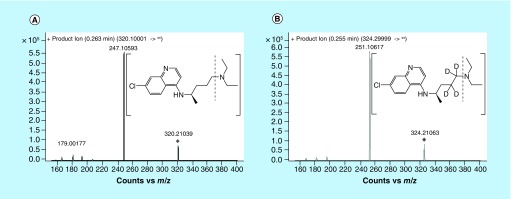
**Product ion spectra of (A) chloroquine, and (B) [^2^H_4_]-chloroquine (IS).**

**Figure 2.**
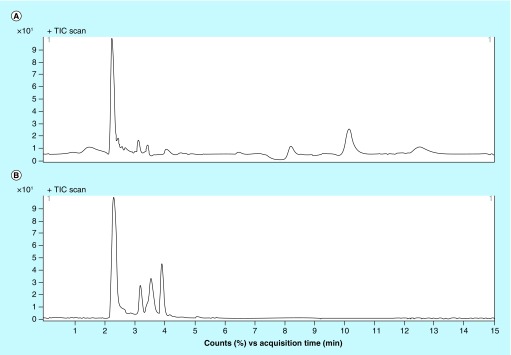
**TIC chromatograms for blank miniature pig precipitated using methanol (A) and acetonitrile (B).**

**Figure 3.**
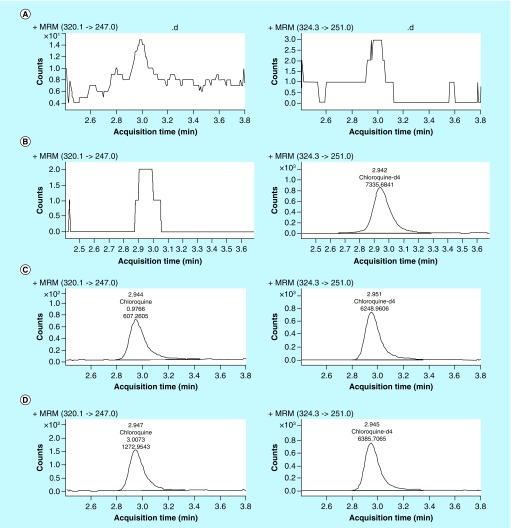
**Representative chromatograms for blank miniature pig plasma (A); blank miniature pig plasma spiked with 40 ng/ml of IS (B); blank miniature pig plasma spiked with 1 ng/ml chloroquine (LLOQ) and 40 ng/ml of IS (C); real miniature pig plasma of 120 h after oral administration of 0.2 g chloroquine formulated in gel (D).**

**Figure 4.**
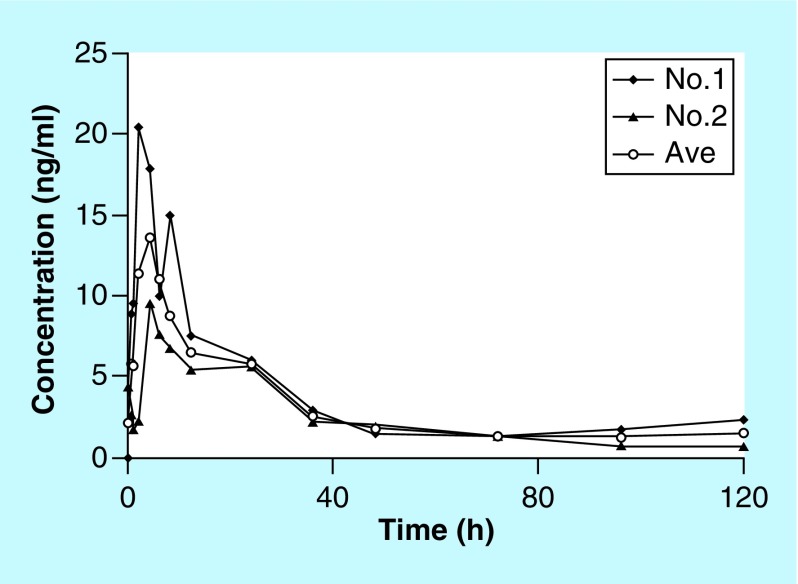
**The mean concentration–time profiles in healthy miniature pigs after oral administration of 0.2 g chloroquine.**

First draft submitted: 22 December 2015; Accepted for publication: 14 April 2016; Published online: 23 May 2016

## Background

Chloroquine (*N*’-(7-chloro-4-quinolyl)-*N,N*-diethyl-pentane-1,4-diamine) was introduced more than 50 years ago for the prophylaxis and treatment of malaria. Except in special patient groups, the use of this inexpensive drug has gradually declined because of widespread resistance and the risk of mortality with overdoses. Chloroquine is now only used for malaria therapy in less developed countries or as a comparator for preclinical testing [1]. Although it has lost its preeminent position in malaria therapy, there is ample clinical evidence that chloroquine, and its derivative hydroxychloroquine, are effective for the treatment of lupus erythematosus, even during pregnancy. Chloroquine and its analogs have also been shown to modify cell differentiation and induce apoptosis in different cancer cell lines, giving hope that they will prove useful adjuncts to cancer chemotherapy [2]. As an autophagy inhibitor, chloroquine co-medication strongly enhanced *in vitro* tumor proliferation rate of docetaxel-loaded PEG-b-PLGA micelles [3], and it also played as a chemical sensitizer in doxorubicin and chloroquine coencapsulated liposomes [4]. Chloroquine gels have been developed as microbicides against HIV-1 infection and exerted a dose-dependent anti-HIV-1 activity *in vitro* [5]. Because of the potential for broader clinical use, there is likely to be ongoing development of chloroquine, and hence a need for a more rapid and efficient detection method for monitoring drug concentrations and preclinical research.

Although many analytical methods have been developed for the detection of chloroquine, most of these use expensive solid phase extraction (SPE) columns [7–9] or require time-consuming liquid–liquid extractions (LLE) combined with extra preacidification operation [10–12] and/or prealkalization [10–13] procedure and then processed to an ultraviolet detector [7–13]. Brief summary of existing HPLC methods for the analysis of chloroquine since 2005 have been concluded and presented in Table 1. Singhal P *et al*. [14] published an LC–MS/MS method for the detection of chloroquine in dog plasma for the first time. A structural analog instead of deuterated isotope internal standard (IS) and a simple protein precipitation by methanol were employed in this study. A traditional reversed-phase C18 (RP18) column was combined with regular mobile phase containing methanol–water (75:25, v/v, 2.5 mM ammonium acetate, pH 4.6). The analysis method for chloroquine was quick, simple and sensitive. However, the absolute recovery of the one-step protein precipitation extraction was unusually rather low (˜19%), which could be related to the matrix effect (not discussed in the assay) since chloroquine could be eluted along with matrixes in the form of salt in current column and mobile phase system. Generally, a traditional RP18 column was hard to reserve solar or basic compounds such as chloroquine. Most of the aforementioned methods used gradient elution [9,13] or relatively high proportion of aqueous phase [8,10–12] to retain the analytes. Still, these methods have obvious drawbacks, including a requirement of large injection volumes (up to 100 μl), long analysis times (especially combined with gradient elution), low sensitivity and limited selectivity, and therefore make it not suitable for fast and robust detection.

HILIC was first introduced by Alpert in 1990 [15] and is an attractive complementary tool for the analysis of small polar/ionic compounds. In addition to the hydrophobic interactions exploited in traditional RP chromatography, HILIC takes advantage of polar partitioning, hydrogen bonding, ion exchange interactions and weak electrostatic interactions, making it especially suitable for the retention and separation of polar compounds [16]. Compared with traditional RP columns, HILIC columns show better retention and more symmetrical peak shapes for basic and polar compounds. HILIC on polar stationary phases typically uses an organic-rich solvent containing 5–40% water as the mobile phase, which enhances MS sensitivity. Because of their versatility, HILIC separations have recently gained popularity for the analysis of polar drugs in complex sample matrices [17].

Chloroquine contains a highly polar tertiary amine that is likely to interact with silanol groups present in the stationary phase of traditional RP columns, usually resulting in poor peak shapes. In almost all published chromatography methods, strong ion-pairing reagents are added to the mobile phase in order to inhibit secondary retention [8,10,11,13], but these buffer salts could cause problems by crystallizing in the desolvation process during MS detection. Since ion-pairing reagents are not required in HILIC [18], HILIC coupled with MS could be an especially suitable tool for analysis of chloroquine.

In the present study, we have developed a rapid procedure, using high-performance HILIC and a one-step method of sample purification by precipitation using acetonitrile, for the determination of concentration of chloroquine in the plasma of miniature pigs. Satisfactory sensitivity and recovery were achieved using highly selective MS with a deuterated IS.

## Experimental

### Standards & chemicals

Chloroquine phosphate was obtained as a powder from the National Institute for Food and Drug Control (Beijing, China, purity 98.9%). [^2^H_4_]-Chloroquine diphosphate salt was purchased from Toronto Research Chemicals (Toronto, Canada) for use as an isotopically labeled IS. The structures of chloroquine and [^2^H_4_]-chloroquine are shown in Figure 1. Chloroquine gel (5%) was manufactured by Guangdong Lewwin Pharmaceutical Research Institute Co., Ltd (Guangzhou, China). HPLC grade methanol and formic acid were supplied by Merck KGaA (Darmstadt, Germany). MS grade ammonium formate was obtained from Sigma-Aldrich Co., LLC (MO, USA). Purified water (conductivity 18MΩ) was obtained using a Milli-Q water purification system (Millipore Corporation, Billerica, MA, USA). Blank miniature pig plasma was kindly provided by Guangdong Lewwin Pharmaceutical Research Institute Co., Ltd and stored at -70°C.

### Instrumentation & conditions

Chromatographic analysis was carried out using an Agilent 1200 HPLC system comprising a G1310A quaternary solvent manager, a G1379B column oven degasser, a G1329A auto-sampler and a G1316A temperature-controlled compartment (Agilent Technologies, Inc., California, USA). Separations were carried out on an HILIC Plus analytical column (100 × 4.6 mm, particle size 3.5 μm, Agilent Technologies, Inc., California, USA) with isocratic elution (mobile phase: 70% methanol, 30% water, 3.5 mM ammonium formate and 0.2% formic acid) at a flow rate of 0.5 ml/min. The injected sample size was 3 μl and the total run time was 3.8 min.

MS detection was performed using an Agilent 6410 triple-quadrupole mass spectrometer (Agilent Technologies, Inc.), operated in positive electrospray ion source (ESI^+^) and multiple reaction monitoring (MRM) mode. The optimized transitions for chloroquine and [^2^H_4_]-chloroquine were *m/z* 320.1 → 247.0 and *m/z* 324.3 → 251.0, respectively.

Agilent MassHunter WorkStation (B.01.03) (Agilent Technologies, Inc., California, USA) was used for both hardware control and data acquisition and processing. Microsoft Office Excel 2007 was used to calculate intra- and inter-assay means, standard deviations, relative standard deviations and coefficients of variation (CV). All PK parameters were calculated using DAS 3.2.4 software (Shanghai University of Traditional Chinese Medicine, China).

### Standard solutions

Stock solutions of chloroquine (1 mg/ml) and IS (0.1 mg/ml) were prepared in 50% methanol and stored at 4°C protected from light. A set of standard solutions were prepared by serial dilution of the stock solutions and used to spike drug-free miniature pig plasma to provide mimic plasma samples with final concentrations of 1, 2, 5, 10, 50, 80 and 100 ng/ml of chloroquine and 40 ng/ml of IS. Quality control (QC) samples were prepared in blank plasma at three levels: low (LQC), medium (MQC) and high (HQC), with concentrations of 3, 10 and 75 ng/ml, respectively.

### Plasma sample preparation

Analytes were extracted by protein precipitation. Briefly, sample (calibration standard, control or test specimen, 100 μl) and IS (20 μl, 200 ng/ml) were placed in an appropriately labeled Eppendorf tube and mixed by vortexing (XW-80A, Shanghai Medical University Instrument Factory, Shanghai, China) for 5 s. Acetonitrile (500 μl) was added to each tube as a precipitant, and the sample was vortexed for 1 min. The mixture was then centrifuged at 14,680 × *g* for 5 min using a Centrifuge 5424 (Eppendorf AG, Hamburg, Germany). Supernatant after centrifugation was transferred into autosamper vials and finally an aliquot of the supernatant (3 μl) was programmed to inject into the HPLCLC–MS/MS system.

### Method validation

The HILIC–MS/MS method was validated based on recommendations by the China Food and Drug Administration and in compliance with the principles of Guidances for Industry Bioanalytical Method Validation by the US FDA and the European Medicines Agency (EMEA). A complete method validation including calibration, carryover, LLOQ, selectivity, accuracy, precision and recovery, dilution integrity (if necessary), matrix effect, reproducibility and stability was carried out.

#### Selectivity, sensitivity & linearity

Selectivity for isotopically labeled IS relative to endogenous matrix (especially phospholipids and inorganic salts) and impurities was tested using six batches of nonpooled, drug-free miniature pig plasma. Samples were prepared and subjected to LC–MS/MS in positive MRM mode as aforementioned description. Peak area for interfering components at retention time of analytes should be invisible or less than 20% of the peak area for lower limit of quantification (LLOQ) for the analyte (1 ng/ml) and 5% for the IS.

The sensitivity of the method was defined by the LLOQ, which was the lowest detectable and reliable concentration of chloroquine within the linear range, with an acceptable degree of precision and accuracy (within ±20%) and signal to noise ratios ≥10-times baseline. The LLOQ was determined by five consecutive injections of prespiked plasma samples (1 ng/ml) and its peak area should be at least five-times the interfering signal of a blank sample.

The calibration curve was designed to respond to likely chloroquine levels in miniature pig plasma following oral administration of the drug. The linearity range of chloroquine was 1–100 ng/ml. A calibration curve was established by plotting the peak area ratios of chloroquine and IS against concentration ratios at seven concentration levels (1, 2, 5, 10, 50, 80 and 100 ng/ml). Calibration curve should be freshly prepared for each analytical batch in method validation and method application process. Double blank sample (processed matrix sample without analyte and without IS) and a blank sample (processed matrix with IS) should be accompanied with each calibration curve to prove the absence of analytes. Double blank and blank samples here should not be included in the calculation of calibration curve parameters. The linearity of the calibration curve was calculated using the least squares regression equation, with a weighting factor of 1/x. A good correlation equation generally requires a coefficient of determination (r^2^) >0.98 and back calculated concentrations of the calibrator should be within ±15% of the nominal value, except for the LLOQ (within ±20%). At least 75% of the calibration and minimum of six calibration standard levels should fulfill this criterion.

#### Precision & accuracy

The precision of the analytical method was processed to discuss the closeness of repeated individual measures and the accuracy was used to determine the accuracy of calibration.

The within-run precisions were investigated at three QC levels (3, 10 and 75 ng/ml) and LLOQ concentration (1 ng/ml) in a single run. As recommended by the EMEA guideline: LQC should be within three-times the LLOQ, MQC around 30–50% of the calibration curve, HQC at least at 75% of the upper calibration curve range. The linearity range of our method was comparatively narrow (1–100 ng/ml), thus MQC of 10 ng/ml was chosen in the method validation. Five samples were contained at each concentration level. Observed peak area ratios were substituted into a fresh accompanying calibration curve to calculate the concentration. The between-run precisions were also evaluated on four concentration levels including LLOQ, LQC, MQC and HQC at least three runs on at least two different days and five samples were included in each level separately. Precision, expressed as the CV, should be within ±15% for LQC, MQC and HQC samples, and ≤20% at the LLOQ concentration.

Within-run accuracy was determined by analyzing five samples per QC level and LLOQ concentration. Observed peak area ratios were substituted into a fresh accompanying calibration curve to calculate the concentration. The back calculated values obtained from the method were compared with the nominal concentrations of the analyte. Deviation from target values was used to present accuracy. The mean concentration bias should be within 15% of the nominal values for the QC samples, except for the LLOQ which should be within 20%.

#### Matrix effect & recovery

Matrix effect and recovery were evaluated in six independent lot of nonpooled and drug-free matrix at three QC concentrations of chloroquine and the operational concentration of IS (40 ng/ml). Using the concept introduced by Matuszewski *et al*. [19], three sample sets were used in our assay: an unextracted sample set, where the analytes were directly diluted using precipitant (that is, pure solution of chloroquine diluted with acetonitrile in this case); a postspiked sample set, where different levels of QC and IS solutions were added to postextracted drug-free biomatrix residues; and a prespiked, extracted set that was prepared as described in ‘plasma sample preparation’ section.

The matrix effect was measured by comparing the peak area ratios of postspiked chloroquine samples at three concentrations to unextracted samples, namely, B/A*100%. As recommended by guidelines, the matrix factor should be calculated for each lot of matrix. IS-normalized matrix factor, the ratio of the matrix effect of the analyte and the matrix effect of the IS was used to quantify the matrix effect in this assay. The matrix effect was investigated at LQC, MQC and HQC concentrations. Consistent values of matrix effect at three analyte levels indicated acceptably reproducible interference by the matrix. The CV of the IS-normalized matrix factor (MF) calculated from the six batches of matrix should not be greater than 15%.

Recovery was measured by comparing peak areas obtained from the extracted samples with those from direct injections of chloroquine solutions at corresponding concentrations, namely, C/A*100%.

#### Carryover

The carryover effect was evaluated by comparing peak areas of three replicates of blank samples, which were injected directly after an ULOQ (100 ng/ml), with a newly injected LLOQ specimen. Generally, a carryover effect no more than 20% of LLOQ and 5% for the IS is regarded as insignificant.

#### Stability

Mimic pig plasma samples at LQC and HQC levels were analyzed along with freshly spiked calibration standards to obtain calculated concentrations. The mean concentration at each level should be within ±15% of the nominal concentration to prove stability at different storage periods.

Stock solution stability was assessed over 40 days at -20°C by comparing the response with that of a freshly prepared chloroquine. Stock solution stability of IS ([^2^H_4_]-chloroquine) was supposed to be similar with that of chloroquine for their structure was nearly the same thus it was not discussed in our research. Benchtop stability was evaluated by allowing QC samples to stand for 6 h prior to preparation. Unextracted QC samples were stored at -20°C for 40 days before extraction to test long-term stability. To analyze freeze–thaw stability, QC samples were stored at -20°C and then allowed to thaw to room temperature for three cycles. Processed QC samples were allowed to stand in the autosampler for 24 h before analysis to determine whether an occasional delay in injection, or reinjection of extracted samples, would lead to degradation. The responses of all QC samples following the different storage conditions and times were substituted into a freshly prepared calibration curve and the mean back calculated concentration at each level should be within ±15% of the nominal concentration to prove stability at different storage periods.

### PK study

The validated LC–MS/MS method was used to investigate the PK profile of chloroquine in Wuzhishan miniature pigs. Two ordinary grade, healthy, Wuzhishan miniature pig (6–8 months old, 7–9 kg) were purchased from Pearl Laboratory Animal Sci. & Tech. Co., Ltd (Dongguan, China) and maintained on standard food and water. The pigs were housed in individual cages and identified by ear tags. Each animal received chloroquine (0.2 g) by oral administration. Blood samples (1.5 ml) were collected in heparinized tubes via the precaval or pelvic vein before medication and 0.5, 1, 2, 4, 6, 8, 12, 24, 36, 48, 72, 96 and 120 h after drug administration. All blood samples were immediately centrifuged at 2000 × *g* for 15 min (Centrifuge 5418, Eppendorf) and then stored at −70°C before analysis. The maximum plasma concentration (C_max_) and time of occurrence (t_max_) were read directly from the concentration-time curve. Other PK parameters were calculated with DAS 3.2.4 software, using a noncompartmental model.

## Results & discussion

### LC–MS method development

Chloroquine has a strongly basic tertiary amine group (Figure 1, pK_a_ 10.47 [20]) that readily interacts with silanol groups in the stationary phase of RPLC columns, probably resulting in secondary retention and consequent broad peak shapes (>1 min). The C18 column was the most frequently used columns in previously published research [7–10,12–14] and we have investigated the peak shape and response of several C18 columns in the optimization process. With additives of 5 mM ammonium formate and formic acid (0.015˜0.1% formic acid) in mobile phase system consisting of acetonitrile and water, chloroquine did not reserve on an Eclipse Plus C18 column (5 μm, 100 × 4.6 mm, Agilent, USA) and the retention time remained almost unchanged (˜1.8 min) with different proportion of acetonitrile ranged between 13 and 70%. The introduction of methanol-water system or acetonitrile-methanol-water system combined with the Eclipse Plus C18 column could not improve the retention behavior (˜2.0 min). The basic compound of chloroquine seems to elute quickly as the form of salts in C18 column and C8 column with the appearance of H^+^ in mobile phase. The remove of H^+^ in mobile phase prolonged the reservation of chloroquine in Eclipse Plus C18 column (2.6 min), ZORBAX SB-C18 column (3.5 μm, 150 × 4.6 mm, Agilent, USA, retention time 3.3–3.5 min), Eclipse XDB-C18 column (5 μm, 150 × 4.6 mm, Agilent, USA, retention time ˜5.5 min) but exerted extremely broad peaks. Since chloroquine contains a highly polar tertiary amine that is likely to interact with silanol groups in the stationary phase of aforementioned RP columns, the deprivation of formic acid in mobile phase possibly related to broad peak shape in our research. Moreover, the wide asymmetrical shapes with long peak trailing, even at high percentages of acetonitrile, suggested the potential for long-term accumulation of analytes on these columns, possibly related to fast consume of columns and unexpected interfering signals in subsequent injections. In almost every published chromatography method, strong ion-pairing reagents were added to the mobile phase to inhibit secondary retention [8,10,11,13], which could cause problems by crystallization of nonvolatile salts in the desolvation process during MS detection. Thus we employed extra additives such as H^+^ to improve peak shape and enhance ionization efficiency in the analysis of chloroquine. We have compared chromatographic behaviors of chloroquine in mobile phase system containing different composition of methanol, acetonitrile, water and formic acid in Proshell 120 EC-C18 column (2.7 μm, 100 × 4.6 mm, Agilent, USA), ZORBAX SB-C18 column and Eclipse XDB-C18 column. Mobile phase consisting of acetonitrile-methanol-water-formic acid exerted narrow but slightly asymmetric peaks (half width about 0.5 min) of chloroquine and the analyte retained weakly in ZORBAX SB-C18 column (retention time 2.8 min) and Eclipse XDB-C18 column (retention time ˜3.3 min). A high proportion of organic solvent containing acetonitrile (˜60%), methanol (˜30%) and 0.1% formic acid (˜10%) gave a comparatively well-shaped peak (half peak width 0.6 min, still broad) on the Agilent XDB column (retention time of 3.25 min) but this still did not meet our requirements. In conclusion, all analytes showed weak retention on these RP columns, regardless of solvent composition, confirming that traditional RP columns are not able to adequately retain chloroquine and that secondary retention was likely to have occurred between analytes and columns.

In many of the published RPLC methods, a strong base such as triethylamine [13,21] or diethylamine [10] was added to competitively conjugate with silanol groups on the stationary phase and thus reduced the binding of amine groups in the analytes with the stationary phase. This procedure, however, remains controversial since it irreversibly changes the character of the column and an alkaline mobile phase is needed to elute the analytes. Some researchers have inhibited secondary retention by increasing the ionic strength of the mobile phase by adding reagents such as perchloric acid [12], phosphoric acid [11] or phosphate buffer [13]. Although this method can be successful with some detection methods, it is not recommended for LC–MS/MS because nonvolatile acids and buffer salts could crystallize during the desolvation procedure and damage the mass spectrometer. In the present study, we tried to optimize the mobile phase and column for LC–MS/MS, and to identify conditions suitable for high-throughput detection. As the proportion of formic acid in the mobile phase was increased to 0.2%, the peak shapes showed some improvement since basic analytes tended to form salts at lower pH and showed less interaction with the stationary phase. High concentrations of formic acid could improve the peak shape, however, coelution of matrix and solvent interfere with detection of the analytes and we chose to use a more polar column to retain chloroquine and other basic analytes as salts. HILIC provides an alternative to HPLC for separating polar compound [17]. As with ion chromatography, for basic compounds such as chloroquine, HILIC gives good peak shapes (no tailing) and reasonable retention times compared with traditional RP columns [22]. Thus an HILIC Plus column (4.6 × 100 mm, 3.5 μm, Agilent) was chosen as separation column in our research.

The optimized flow rate (<0.5 min) for mass spectrometer is generally far less than the optimal flow rate for well separation in LC (˜0.8 min). However, the mass detector generally requires relatively low flow rate for efficient spray, which is a key factor for MS and could strongly impact the signal response here. We employed a compromised flow rate of 0.5 ml/min here and tried to balance the retention and signal response by adjusting mobile phase component. The retention time of chloroquine on underivatized HILIC columns was longer when the mobile phase contained a higher proportion of organic solvent. The retention times of the main matrix components, such as phospholipids, were reported not significantly altered by varying the composition of the mobile phase [16], thus we tried various mobile phase to separate analytes and matrix components, which could be the main contributor of low apparent recovery (˜20%) after one-step protein precipitation in Singhal P's research [14].

As shown in Figure 2, the major matrix components eluted in two distinct groups, with peaks at 2.1–2.6 and 3.1–3.9 min. A mobile phase composed of 70% methanol, 30% water, 3.5 mM ammonium formate and 0.2% formic acid enabled a little retention in the HILIC column and eluted as symmetrical peaks in 2.9 min. Gradient elution could realize better separation between analytes and interfering components whereas gradient elution usually requires balance time as long as half an hour in HILIC columns to obtain steady column pressure. A good balance of retention time, peak shape and matrix effect was achieved on an HILIC Plus column (3.5 μm, 100 × 4.6 mm) and current mobile phase consisting of methanol and water.

Standard solutions of chloroquine (1 μg/ml in methanol) and IS (0.1 μg/ml in methanol) were directly infused into the mass spectrometer to optimize the transitions and operating conditions separately. Product ion splitting spectra of (A) chloroquine, and (B) [^2^H_4_]-chloroquine (IS) was presented in Figure 1. Transitions of *m/z* 320.1 → 247.0 and *m/z* 324.3 → 251.0 were employed to monitor chloroquine and IS, respectively, as summarized in Table 2. The nebulizer gas, auxiliary gas and curtain gas was produced using a Brezza nitrogen generator (Claind srl, Lenno, Italy), and high purity nitrogen was used as the collision gas. The ion spray temperature was 350°C, the ion spray voltage was 4000 V, the drying gas flow rate was 10 l/min and the nebulizer gas pressure was 45 psi. The impact energy and split voltage were 14 units and 135 V, respectively, with dwell time of 200 ms. The electron multiplier was set at 200 units for all samples.

### Extraction method development

Extraction procedures using LLE and SPE with HPLC have been employed to analyze chloroquine in biomatrix previously. Preparation of biological samples by LLE usually results in large injection volumes and needs relatively complicated and time-consuming pretreatment, including acidification [10,11] and basification [7,10,11], whereas SPE procedures require expensive SPE columns and longer analysis times [6–9].

Because of the high sensitivity of MS detection, a simpler and more convenient protein precipitation procedure successfully met the requirements of the present PK study [14]. Different precipitation agents, including methanol and acetonitrile, were evaluated during optimization of the extraction procedure. Total ion chromatography graphs of different precipitant-treated drug-free miniature pig plasma samples are shown in Figure 2. Significant signal peaks and valleys were observed as late as 14 min after injection of the methanol-treated sample, indicating that the matrix components remain on the column and do not elute within one gradient cycle (run time 3.8 min). Samples treated with acetonitrile gave a lower and smoother baseline than those treated with methanol and there was less carryover of impurities to the next run.

We have compared different mobile phase system containing acetonitrile and water and observed similar peak shape as the methanol-water system. It seems it is the type of column rather than composition of mobile phase or sample solvent that impact the peak shape. Thus peak incompatibilities between the sample solvent of acetonitrile and mobile phase consist of methanol and water didn't result in peak distortion. Acetonitrile was, therefore, chosen as the precipitant for less carryover of impurities and efficient one step precipitation in our assay.

## Method validation

### Specificity, sensitivity, linearity & carry over

The specificity of the method was evaluated using chromatograms of blank miniature pig plasma. Typical MRM chromatograms of blank and spiked miniature pig plasma are presented in Figure 3. The retention times for both chloroquine and IS were approximately 2.9 min in the LC–MS/MS trace, and there were was no observable matrix component at the retention time of analytes. Satisfactory sensitivity was achieved in the assay, with a signal to noise ratio >20, and precision and accuracy values between −5.2 and 6.8% at LLOQs.

Correlation coefficients (R^2^) were >0.99 for all routine calibration curves, indicating a high degree of correlation between y (response ratio) and x (concentration ratio) values over the range 1–100 ng/ml. A linear 1/x weighting regression was used. The pooled slope and intercept were 1.7611 ± 0.0574 and 0.0409 ± 0.0023, respectively. Calibration accuracy for all seven calibrators showed less than ±15% bias and no carry over was observed in blank samples after injections containing 100 ng/ml chloroquine.

### Matrix effects & recovery

The matrix effect was evaluated to reveal possible suppression or enhancement of ionization by associated endogenous components in the biological matrices. The peak response ratios of chloroquine and IS in postextracted samples were compared with those in solvent samples of equivalent concentration and MFs from six lots were summarized in Table 3. The mean IS-normalized MFs were ranged between 106.00 and 116.81%, and the CV of the IS-normalized MF calculated from the six lots of matrix was less than 5%, indicated negligible effects of endogenous matrix on analyte ionization under the current validation conditions.

The absolute recoveries (also known as overall process recoveries) of chloroquine at three QC levels (3, 10 and 75 ng/ml) were 92.32 ± 10.86%, 95.57 ± 3.63% and 97.57 ± 5.07%, respectively. The mean recovery of IS was 95.49 ± 5.81%.

### Precision & accuracy

Five replicates of plasma samples were used to measure the precision and accuracy for chloroquine. The within-run precision had a CV of 1.56–11.33% at different levels and the between-run precision was 0.82–18.47% (Table 4). The deviation from nominal value of within-run ranged from −0.21 to 6.10% and between-run bias was from 0.15 to 6.4%. Accuracy and precision values were within the guidelines (%CV and bias <20% for LLOQ and <15% for LQC, MQC and HQC), demonstrating the reliability and reproducibility of the assay.

### Stability

The stability of chloroquine is summarized in Table 5. Recovery from prepared samples in miniature pig plasma indicated that chloroquine was stable for at least 6 h during bench top preparation and for at least 40 days storage at −20°C. Prepared samples were also found to be stable after storage in the autosampler for 24 h. Miniature pig plasma samples containing chloroquine can thus be prepared and used under laboratory conditions without significant degradation of the chloroquine.

### Method application

The newly developed analytical method for chloroquine was used to quantify plasma concentrations in a PK study. Mean concentration-time profiles of chloroquine after a single oral dose (0.2 g) in healthy miniature pigs are shown in Figure 4. Average C_max_, t_max_, elimination t_1/2_, CL/F, V/F and AUC_inf_ values for chloroquine were 15.03 ng/ml, 3 h, 16.9 h, 54.15 l/h, 1543.82 l and 380.02 ng/ml*h. The method was sufficiently sensitive to monitor plasma concentrations up to 120 h. Calibration and QC samples were well within acceptable limits. As far as we know, this is also the first report on PK profile of chloroquine in miniature pigs and further comparison of the PK results might be required to confirm the application of current method.

## Conclusion

A novel, fast and sensitive LC–MS/MS method for the determination of chloroquine has been developed and validated in miniature pig plasma. An HILIC column was employed to separate the analytes and gave symmetrical peak responses. The method employed a one-step protein precipitation procedure using acetonitrile and a sensitive detection method using a MassHunter Work Station. A total run time of 3.8 min/injection and negative carry over after a 3.0 μl injection met the requirements for routine high-throughput analysis. Validation results demonstrated good selectivity, high sensitivity, accuracy and precision over the concentration range of interest, as well as appropriate recovery and lack of matrix interference. The robustness of the assay was confirmed by a PK study in miniature pigs.

## Future perspective

The development of fast and sensitive LC–MS/MS methods for the determination of chloroquine is essential to explore new applications for this old-fashioned drug. HILIC is a promising alternative to RPLC, especially for highly polar/ionic compounds. We have developed a simple, highly sensitive and rapid method for the determination of chloroquine and used this method for high-throughput analysis of chloroquine in a PK study. Future works should however address issues such as flow rate, which in the present method is far less (0.5 ml/min) than the optimal flow rate in LC, to achieve balance retention time and efficient spray. We believe a postcolumn flow diverter could improve the situation in some degree. In addition, since only two pigs were included in the PK study, the sample size is too small for statistical analysis. As such an increased number of test animals are needed in subsequent research.

That said, with only a change of mobile phase to avoid possible matrix effects, our new method could be adapted for detection of chloroquine in other biological fluids. Lower LLOQs and faster detection speeds could be achieved using advanced instruments to meet the demands of clinical research.

**Table 1.** **Brief summary of HPLC and LC–MS/MS methods for the detection of chloroquine since 2005.**

	**Our assay**	**Singhal [14]**	**Zuluaga-Idarraga [13]**	**Cheomung [10]**	**Lejeune [9]**	**Deng [8]**	**Samanidou [7]**	**Yonemitsu [11]**
Internal standard	Chloroquine-*d4*	Piperazine bis chloroquinoline	/	Quinine	Bisdemethyled derivative of cycloguanil	8-chloro-4-aminoquinoline	Salicylic acid	Strychnine nitrate

Volume for processing (ml)	0.1	0.05	0.5	0.15	0.08	1	0.04 (blood serum), 0.1 (urine)	1

Preparation process	Precipitation	Precipitation	LLE, prebasified	Acidized, basified and then processed to LLE	SPE	SPE	SPE	LLE, prebasified and acidized

Selection of precipitant or extractant	Acetonitrile	Methanol	*n*-hexane organic solvent and ethyl acetate (57.5:42.5, v/v)	Hexane and *tert*-butyl methyl ether (1:1, v:v)	/	/	/	Ether

Injection volume (μl)	3	10	100	80	70	20	50	20

Column	HILIC Plus column (3.5 μm, 100 × 4.6 mm)	Chromolith SpeedROD C18 column (50 × 4.6 mm)	BDS Hypersil C18 column (5 μm, 250 × 4.6 mm)	Thermo Hypersil Gold C18 column (5 μm, 250 × 2.1 mm)	X-Terra C18 column (5 μm, 100 × 4.6 mm)	X-terra C18 column (5 μm, 250 × 4.6 mm)	MZ Kromasil C18 (5 μm, 250 × 4 mm)	TSK gel Super-ODS column (100 × 4.6 mm)

Mobile phase	Methanol–water (70:30, v/v, 3.5 mM ammonium formate and 0.2% formic acid)	Methanol–water (75:25, v/v, 2.5 mM ammonium acetate, pH 4.6)	Gradient elution. Aqueous phase: orthophosphoric acid (0.57%), sodium hydroxide (0.087 M) and triethylamine (0.13 mM), pH 2.70. Organic phase: methanol	1% diethylamine–acetonitrile–methanol (20:55:25, v:v:v)	Gradient elution. (A) acetonitrile–potassium phosphate (pH 5.5, 40 mM) (12:88, v/v) and (B) acetonitrile–potassium phosphate (pH 5.5, 40 mM) (40:60, v/v)	Acetonitrile–20 mM borate buffer (40:60, v/v)	Methanol–acetonitrile–0.1 mol/l ammonium acetate (45:15:40 v/v)	Acetonitril–20 mM 1-heptanesulfonic acid containing 0.07% diethylamine (pH 3.4, 30:70, v/v)

Retension (min)	2.9 (3.8)	1.1 (2.0)	6 (14)	6.4 (10)	10.4 (35)	20 (28)	3.5 (7)	5.6 (10)

absolute recovery	92.32–97.57	18.6–19.0	73–85.4	79.99–95.33	85–89	101–102	105.4 for serum and 90.7 for urine (relative extraction recovery)	79 ± 5

LLOQ (ng/ml)	1	2	6.4	25	150	80	300	50

Linear range (ng/ml)	1–100	2.0–489.1	20–2000 nM	25–1500	150–2500	250–2000 nM	300–500	50–10,000

Application	Pig plasma	Dog plasma	Human plasma	Patient whole blood	Human whole blood	Human whole blood	Human serum and urine	Human blood and solid tissue

**Table 2.** **LC–MS/MS parameters selected for the quantification of chloroquine using [^2^H_4_]-chloroquine as internal standard.**

**Analyte**	**Q1^†^**	**Q3^‡^**	**Dwell (ms)**	**Fragmentor (eV)**	**Collsion energy (eV)**
Chloroquine-*d4*	324.3	251	200	145	16

Chloroquine	320.1	247	200	145	16

^†^Precursor ion (*m/z*).

^‡^Product ion (*m/z*).

**Table 3.** **Matrix effect for the detection of chloroquine.**

**Nominal conc. (ng/ml)**	**Lot 1**	**Lot 2**	**Lot 3**	**Lot 4**	**Lot 5**	**Lot 6**
Matrix effect (mean ± SD, %)	106.10 ± 0.38	108.48 ± 1.68	106.74 ± 0.36	105.52 ± 1.86	115.27 ± 7.97	111.59 ± 6.22

IS normalized matrix effect (mean ± SD, %)	106.00 ± 1.34	106.49 ± 2.48	106.91 ± 1.78	105.64 ± 2.22	113.37 ± 9.57	116.81 ± 13.36

**Table 4.** **Validation results for within-day precision and between-day precision.**

	**Within-run (n = 5)**				**Between-run (n = 15)**			
Nominal conc. (ng/ml)	1	3	10	100	1	3	10	100

Back-calculated conc. (mean ± SD, ng/ml)	1.00 ± 0.07	3.18 ± 0.13	10.44 ± 0.47	74.86 ± 1.02	1.00 ± 0.11	3.19 ± 0.17	10.21 ± 0.44	75.17 ± 1.09

Precision (CV, %)	11.33	5.85	4.70	1.56	18.47	9.16	4.75	0.82

Accuracy (%)	99.79 ± 6.70	106.10 ± 4.45	104.37 ± 4.68	99.81 ± 1.36	100.15 ± 10.51	106.40 ± 5.77	102.12 ± 4.45	100.23 ± 1.45

**Table 5.** **Stability of chloroquine in solution and miniature pig plasma under different storage conditions.**

**Condition**	**LQC (3 ng/ml)**	**HQC (75 ng/ml)**
**Solution stability (40 day)**		

Recovery (mean ± SD, %)	101.74 ± 6.75	102.6 ± 2.46

**Long-term stability (40 day)**		

Recovery (mean ± SD, %)	105.99 ± 3.35	104.27 ± 1.04

**Freeze–thaw stability (3 cycles)**		

Recovery (mean ± SD, %)	106.02 ± 9.74	105.63 ± 1.07

**Benchtop stability (8 h)**		

Recovery (mean ± SD, %)	104.16 ± 1.33	103.49 ± 1.00

**Processed stability (24 h)**		

Recovery (mean ± SD, %)	98.90 ± 1.08	105.70 ± 0.34

**Executive summary**

**Background**

- The old-fashioned drug of chloroquine has been a promising candidate in cancer chemotherapy and HIV infection, but a quick and reliable detection method is still limited.

**Method optimization**

- Several reversed-phase columns were compared and an HILIC column was chosen to separate chloroquine in miniature pig plasma using methanol-water mobile phase system. Ammonium formate and formic acid were added to mobile phase to obtain good peak shape and reduce matrix effect. An efficient one-step precipitation extraction by acetonitrile was employed to obtain satisfactory sensitivity.

**Method validation**

- The method has been fully validated according to regulatory guidelines, including calibration, carryover, sensitivity, selectivity, accuracy, precision and recovery, matrix effect, reproducibility and stability.

**Method application**

- The method has been successfully used in a PK study in miniature pigs.
